# Is the management of acute confusional syndrome secondary to covid-19 pneumonia different from the management of confusional syndrome secondary to other causes?

**DOI:** 10.1192/j.eurpsy.2022.787

**Published:** 2022-09-01

**Authors:** A. Llimona González, M. Calls Samora, D. García Hernández, S. Oller Canet

**Affiliations:** Parc de Salut Mar, Instituto De Neuropsiquiatría Y Adicciones (inad), Barcelona, Spain

**Keywords:** delirium, ACUTE CONFUSIONAL COVID-19, CONFUSIONAL, Covid-19

## Abstract

**Introduction:**

Acute Confusional Syndrome (ACS) is the most common neuropsychiatric complication in COVID-19 infection. Its management is still a challenge because the data and recommendations based on the evidence are limited.

**Objectives:**

To describe the differential characteristics in the management of ACS in patients with COVID-19 pneumonia compared to ACS secondary to other causes.

**Methods:**

We present a descriptive study that is has been carried out in 62 patients with ACS (26 of them diagnosed with COVID 19 pneumonia), who have required assessment by the liaison psychiatry service of Hospital del Mar between February and April, 2020. The sample was divided in 2 groups (with and without COVID 19 pneumonia). Chi square and Fisher’s tests were used to comparisons.

**Results:**

Dexmetomidine (26 vs 0) and olanzapine (13 vs 3) were significantly more frequently used in COVID-19 patients (p< 0 001). A greater number of different antipsychotic drugs were used in COVID 19 patients (2.40± 1 323 number of drugs), (p<0.0001). Further neuroimaging tests were requested in COVID 19 patients and they received less family support (4) compared to non COVID-19 (22), (p<0.005).

**Conclusions:**

ACS associated with COVID-19 pneumonia in the patients in our sample is more difficult to manage than ACS associated with other pathologies, similar to which described in other series. It is associated with a longer duration of confusional symptoms and difficulties for control it.

**Disclosure:**

No significant relationships.

